# Microvascular Complications of Food Insecurity in a Vulnerable Patient Population

**DOI:** 10.3390/diseases14070250

**Published:** 2026-07-11

**Authors:** Morgan Uebinger, Megan Gremillion, Stephanie M. Provenzano, Carliss Sampognaro, Lindsey Settoon, Emma Mayfield, Mason Granger, Courtlin Wadleigh, Ahmed I. Anwar, Alan D. Kaye, Ammar Husan

**Affiliations:** 1 Department of Family Medicine, Louisiana State University Health Shreveport, 1501 Kings Highway, Shreveport, LA 71103, USA; 2 Department of Anesthesiology, Louisiana State University Health Shreveport, 1501 Kings Highway, Shreveport, LA 71103, USA

**Keywords:** food insecurity, microvascular complications, diabetes mellitus, retinopathy, neuropathy, nephropathy, inflammation

## Abstract

**Background:** Food insecurity (FI), which is defined as limited or uncertain access to sufficient, nutritious food, disproportionately affects low-income populations and has been linked to poor control of chronic diseases including type 2 diabetes mellitus (T2DM) with North Louisiana having one of the highest rates of FI in the United States. The interaction between FI and neighborhood-level socioeconomic disadvantage has not been well characterized in relation to microvascular diabetic complications. **Objective:** This study aimed to evaluate the relationship between FI, socioeconomic disadvantage, and microvascular complications in patients with T2DM. **Methods:** A retrospective cohort study of 163 adult patients with T2DM who received care at Ochsner LSU Health clinics in Shreveport and Monroe, Louisiana, between January 2018 and January 2023 were reviewed. FI status, Area Deprivation Index (ADI), a validated measure on neighborhood socioeconomic disadvantage, and microvascular complication outcomes including nephropathy, neuropathy, and retinopathy were obtained from electronic medical records. FI was assessed using the validated two-item Hunger Vital Sign screening tool. **Results:** The study found that FI alone was not a statistically significant predictor of nephropathy based on microalbumin-to-creatinine ratio (MACR). However, the combination of FI and high ADI (≥7) was significantly associated with neuropathy (*p* = 0.024) and retinopathy (*p* < 0.001), particularly among patients with T2DM for more than five years. Additionally, the interaction between FI and high ADI was also found to influence MACR values significantly (*p* = 0.026), indicating that socioeconomic stressors may exacerbate kidney dysfunction. **Conclusions:** Food insecurity, coupled with socioeconomic adversity, was associated with an increased risk of microvascular complications in T2DM. These results highlight the importance of integrating FI screening and addressing social determinants of health in high-risk populations. Early identification and targeted interventions could help mitigate disease progression and reduce health disparities.

## 1. Introduction

Patients experiencing FI often suffer from hypertension, heart disease, diabetes, kidney disease, chronic obstructive pulmonary disease (COPD), cancer, and stroke, among others, at a higher rate than food-secure persons [1]. Poor health status can increase economic disparities in patients with chronic conditions, such as type II diabetes mellitus (T2DM), due to higher healthcare treatment expenditures [1]. The Area Deprivation Index (ADI) is a measure of disparity by social factors and is detailed geographically to better describe community situations of disparity and the interplay between these factors [2]. Overall, ADI provides additional context for patients’ health and can assist in directing patient-centered care practices. The combined impact of individual-level food insecurity and neighborhood-level socioeconomic deprivation on chronic disease outcomes, including diabetic microvascular complications, remains insufficiently characterized.

Additionally, FI often coexists with other social determinants of health (SDOHs), such as socioeconomic disparities, housing instability, and lack of healthcare access [3,4]. Such social factors can exacerbate challenges faced by those experiencing FI.

Louisiana has one of the highest rankings for FI in the nation, with 16.9% of Louisianians experiencing FI in 2022 compared to the national average of 11.7% [5,6]. Louisiana also ranks in the top 20% for states with a high ADI, representing a significant rate of socioeconomic disadvantage. Additionally, Louisiana sits at or above the national average for the diagnosis of T2DM, emphasizing the high prevalence of the disease [7]. Of note, studies have found a significant increase in the prevalence of diabetes in Louisiana neighborhoods with high ADIs [7]. North Louisiana is particularly affected by FI, with 16.2% of Caddo Parish residents and 17.2% of Ouachita Parish residents experiencing difficulty in consistently obtaining nutritious foods [8]. These two parishes are especially relevant to researchers as they are home to Ochsner LSU Health hospitals and clinics in North Louisiana [6]. High rates of FI and T2DM in Louisiana highlight a vulnerable patient population, as inadequate access to nutritious food has a profound impact on the development and management of chronic conditions [8].

The current literature has extensively explored the associations between FI and T2DM [9]. T2DM management involves dietary modifications, such as carbohydrate counting, limiting added sugars, increasing protein, and boosting fiber intake; however, individuals facing FI struggle to obtain nutritious foods, making adherence to a diabetic diet extremely challenging [10,11]. A 2022 study found that not having enough food and not having access to high-quality foods are associated with higher HbA1c levels, suggesting FI and diet quality are crucial to glycemic control [12]. The financial burden of managing diabetes for patients with FI and other SDOHs (e.g., paying for medications) often competes with buying food, forcing patients to choose between acquiring the necessary medications and the food crucial to fueling their bodies [11]. These complex interactions are thought to magnify the burden of T2DM in FI patients, hastening the development of diabetic-related complications [13]. Beyond glycemic control, T2DM is recognized as a chronic inflammatory state characterized by insulin resistance, oxidative stress, endothelial dysfunction and vascular injury [14]. These inflammatory and metabolic abnormalities contribute to the development of diabetic microvascular complications which include nephropathy, and in this context, FI may plausibly intensify microvascular risk by worsening diet quality and the broader inflammatory burden associated with diabetes [14].

As a consequence of longstanding, uncontrolled T2DM, many patients will eventually develop microvascular complications [15]. These complications are not solely a consequence of hyperglycemia but also reflect chronic inflammation, oxidative stress, and endothelial damage [16]. Diabetic neuropathy, for example, is influenced by the duration of disease and elevated HbA1c levels [17]. While obesity and metabolic complications, such as high triglyceride levels and low high-density lipoproteins, have been shown to exacerbate diabetic neuropathy, there are limited data on the association between food security status and the development of neuropathy [17,18,19]. Another example of a microvascular complication of T2DM is diabetic retinopathy [15]. Diabetic retinopathy is caused by high blood glucose levels damaging the retinal blood vessels, which can lead to vision loss or blindness [20]. It has been linked to patients who experience FI as they are less likely to access necessary care associated with the prevention and treatment of retinopathy, leading to earlier onset and more rapid progression [21]. However, there are limited data regarding the complex interplay between FI and other SDOHs on the development of neuropathy and retinopathy in T2DM, as these patients are less likely to get proper screening with yearly diabetic eye and foot exams and often have worse glycemic control [22].

Additionally, the association between chronic kidney disease (CKD) and T2DM is well known, with roughly 40% of T2DM patients developing nephropathy as the disease progresses [23]. Diabetic nephropathy describes physical changes to the kidney due to the detrimental effects of longstanding hyperglycemia [24]. A 2014 study examining the effect of FI on CKD in low-income Americans found that FI is associated with a greater chance of developing nephropathy in diabetic individuals [25]. Patients with nephropathy who experience FI are also more likely to progress to end-stage renal disease (ESRD) compared to food-secure individuals [26]. Further, FI has been shown to exacerbate nephropathy as these patients are often unable to adhere to a specific kidney-protective diet due to the intricate dietary details balancing minerals and electrolytes [26,27,28]. As with diabetic retinopathy and neuropathy, additional studies are needed to quantify the additive effects of FI among other SDOHs and socioeconomic disparities on diabetic nephropathy.

FI affects millions annually and has been associated with the development and exacerbation of chronic health conditions, such as T2DM, and this study aims to evaluate the association between FI and the development of microvascular complications (neuropathy, retinopathy, and nephropathy) in a vulnerable population with high rates of insufficient access to nutritious food [1,4]. The authors hypothesize that FI patients will experience a greater burden of microvascular complications, with a stronger interaction in those experiencing additional socioeconomic stress.

## 2. Methods

This retrospective cohort analysis was conducted across five years (January 2018–January 2023) among adult T2DM patients aged 20–75 (*n* = 170), with 7 excluded due to incomplete key variables through a complete-case approach, including relevant information regarding FI status, age, ADI, or if they were in a marginalized population including pregnant women, incarcerated persons, and cognitively impaired patients. These patients were receiving medical care from Ochsner LSU Health clinics and hospitals in Shreveport and Monroe, Louisiana. Data were extracted from the Epic Electronic Medical Record (EMR) via chart review by research associates. The data that were extracted included the following: FI status, age, race, ethnicity, sex, Area Deprivation Index (ADI) state decile, smoking status, microalbumin-to-creatinine ratio (MACR), hemoglobin A1C (HbA1c), glomerular filtration rate (GFR), T2DM status, duration of T2DM, T2DM medications, and microvascular complications of T2DM, including nephropathy, retinopathy, and neuropathy. FI status was determined via responses to the Hunger Vital Sign (HVS) questionnaire, a validated two-question survey validated by the American Academy of Pediatrics and Feeding America [29]. FI status was assessed at the time of clinical encounter and reflects a single time point. Additionally, ADI is a validated tool that provides insight into socioeconomic disadvantage experienced at the individual and community levels [2]. ADI was analyzed using a cutoff of ≥7 to represent a higher neighborhood-level socioeconomic disadvantage consistent with study grouping practices, with the prior literature emphasizing that area deprivation measures are context-dependent and should be operationalized based on study design and the populations characteristics [30]. T2DM status was determined by the International Classification of Diseases, Tenth Revision, Clinical Modification (ICD-10-CM) code E11. T2DM-related neuropathy was defined as ICD-10-CM codes E11.40 and E11.42; T2DM-related retinopathy was defined as ICD-10-CM codes E11.30, E11.31, E11.32, E11.33, E11.34, and E11.35. Microvascular complications were identified through documented ICD-10-CM codes within the EMR, and because EMR-based diagnosis capture depends on clinical documentation and screening practices, the presence of complications may reflect differences in healthcare access and frequency of evaluation. MACR, an early indicator of diabetic nephropathy, was categorized into three groups indicating disease severity: normal (<30 mc/g), microalbuminuria (30–300 mc/g), and macroalbuminuria (>300 mc/g).

Dates of MACR, hemoglobin A1c, and GFR collection fell within 3 months of one another. T2DM-related medications were reviewed and reported if they were administered within 3 months of the MACR, hemoglobin A1c, and GFR test. Analysis using Jamovi (Version 2.6.26) included descriptive statistics, normality tests via Shapiro–Wilk, T-tests, ANCOVAs, linear regressions, Chi-squared tests (*X*^2^) with a degree of freedom (df) of 1, and odds ratio (OR) calculations within a 95% confidence interval (CI). Chi-squared analyses were used to evaluate unadjusted associations. Multivariable models were used where applicable to account for confounding by clinically relevant covariates available in the dataset, including age, sex, race/ethnicity, ADI, smoking status, and duration of T2DM. Data in violation of normality per Shapiro–Wilk (*p* < 0.05) were transformed via base-10 logarithm. This study and subsequent analysis were approved by the Louisiana State University (LSU) Health Shreveport School of Medicine Institutional Review Board (IRB) on 27 September 2024, referenced as STUDY00002796. The study adheres to the Declaration of Helsinki. The final analytic sample (N = 163) reflects exclusion of records with incomplete key variables.

## 3. Results

### 3.1. Demographics of the Study Population

The dataset included a total of 163 patients, 46% of whom experienced FI and 54% who did not. Demographic characteristics and smoking status of the sample size are displayed in Table 1. A majority of the patients were female (52.76%), Black or African American (AA) (66.26%), not Hispanic or Latino (99.39%), between the ages of 61 and 70 (38.04%), and had no smoking history (50.92%). Most patients fell within an ADI state decile of 9 or 10 (50.92%), indicating a high level of socioeconomic disadvantage at the neighborhood level. The mean age for the dataset was 58, and the mean ADI was 7.71.

### 3.2. Food-Insecure Patients Demonstrated Differences in Microalbumin–Creatinine Ratios and GFR Compared with Patients That Are Food-Secure

The mean values for MACR, hemoglobin A1c, and GFR, as well as duration of T2DM among participants in the dataset with and without FI, are detailed in Table 2. Mean values for MACR and GFR were numerically greater in FI than in non-FI patients, while mean hemoglobin A1c values and duration of disease were numerically greater in patients who did not experience FI.

### 3.3. Food Insecurity and High ADI Demonstrated an Interaction Associated with Differences in Microalbumin–Creatinine Ratios

**Figure 1:**
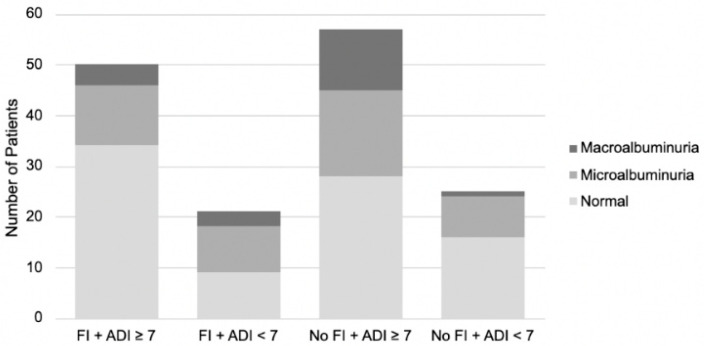
Distribution of microalbumin-to-creatinine ratio (MACR) categories (normal, microalbuminuria, and macroalbuminuria) across combined food insecurity (FI) and Area Deprivation Index (ADI) groups. Patients are stratified into four categories: FI with ADI ≥ 7, FI with ADI < 7, non-food-insecure (No FI) with ADI ≥ 7, and No FI with ADI < 7. ADI is categorized using state deciles (low < 7, high ≥ 7). The y-axis represents the number of patients within each subgroup. FI = food insecurity; ADI = Area Deprivation Index.

MACR categorization as an indicator for the degree of diabetic nephropathy within the dataset regarding FI status and ADI is shown in Figure 1. Among FI patients with a known ADI (*n* = 71), 70.42% possessed an ADI ≥ 7, which is indicative of a high degree of socioeconomic disadvantage. The MACRs of FI patients with an ADI ≥ 7 were mainly within normal limits (68.00%), followed by microalbuminuria (24.00%) and macroalbuminuria (8.00%) (Figure 1). Of FI patients with an ADI < 7, 42.86% of MACR values were within normal limits, 42.86% had microalbuminuria, and 14.29% had macroalbuminuria (Figure 1). Most food-secure patients with an ADI ≥ 7 had MACR values within normal limits (49.12%), followed by microalbuminuria (29.82%) and macroalbuminuria (21.05%) (Figure 1). In contrast, in food-secure patients with an ADI < 7, MACR values were mainly within normal limits, followed by 32% with microalbuminuria and 4% with macroalbuminuria (Figure 1). Thus, higher proportions of macroalbuminuria were observed in food-secure patients with an ADI ≥ 7; however, these subgroup patterns should be interpreted in the context of the significant FI X ADI interaction, indicating that the association between FI, ADI, and MACR was not uniform across strata. This counterintuitive finding suggests that relationship between FI, neighborhood-level deprivation, and albuminuria may be influenced by additional clinical or demographic factors not fully captured in the current dataset.

**Figure 2:**
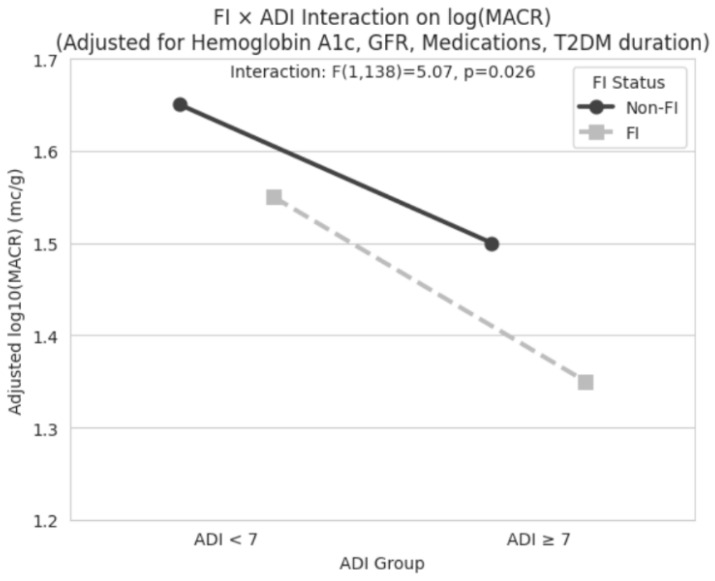
Interaction between food insecurity (FI) status and Area Deprivation Index (ADI) in predicting log-transformed microalbumin-to-creatinine ratio (log10 MACR), adjusted for hemoglobin A1c (HbA1c), estimated glomerular filtration rate (GFR), medication use, and duration of type 2 diabetes mellitus (T2DM). The y-axis represents adjusted log10(MACR) values (mg/g), and the x-axis represents ADI category (low < 7 vs. high ≥ 7 state deciles). FI status is stratified into food-insecure (FI; dashed line) and non-food-insecure (No FI; solid line) groups. The interaction between FI and ADI was statistically significant (F [1,138] = 5.07, *p* = 0.026).

Initially, MACR was not normally distributed (Shapiro–Wilk W = 0.377, *p* < 0.001); thus, logarithmic transformation (base 10) was utilized for analysis. Per independent-samples T-test, no statistically significant differences were observed across log (MACR) (t [159] = 1.14, *p* = 0.257), hemoglobin A1c (t [160] = 0.928, *p* = 0.355), or GFR (t [159] = −0.570, *p* = 0.570) between FI and non-FI patients. However, after adjusting for hemoglobin A1c, GFR, medication use, duration of T2DM, and ADI, an ANCOVA revealed a significant FI and ADI interaction associated with log (MACR) (F [1,138] = 5.07, *p* = 0.026) (Figure 2). However, FI alone was not independently associated with log (MACR) (F [1,138] = 3.09, *p* = 0.081). Given a modest sample size, greater emphasis was placed on effect sizes, confidence intervals, and *p*-values, when interpreting the findings.

### 3.4. Food Insecurity and ADI Demonstrated Differing Patterns of Association with MACR

When analyzing the interaction between FI and log-transformed MACR, the non-parallel orientation of the lines in Figure 2 suggests a significant interaction (*p* = 0.026). In both high (ADI ≥ 7) and low (ADI < 7) groups, log10(MACR) decreased as ADI increased. In FI patients (light grey, dashed line), the slope appears steeper, suggesting a more substantial association of ADI with log (MACR) compared to food-secure patients (dark grey, solid line) (Figure 2). The mean of log (MACR) for FI patients from high- and low-ADI groups was 1.35 mc/g and 1.55 mc/g, respectively (Figure 2). The mean of log (MACR) for food-secure patients from high- and low-ADI groups was 1.50 mc/g and 1.65 mc/g, respectively (Figure 2), indicating that within this sample, food-secure patients in low-ADI groups demonstrated higher mean log (MACR) values, highlighting the complexity of the relationship between FI, ADI, and albuminuria.

### 3.5. Food Insecurity and High ADI Demonstrated Differences in the Prevalence of Microvascular Complications

**Figure 3:**
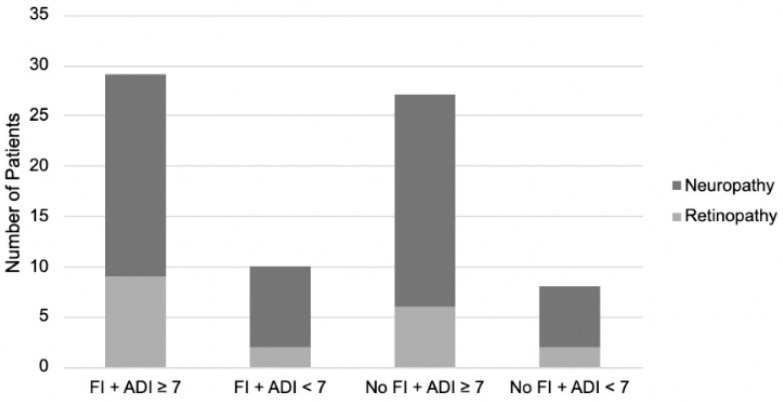
Prevalence of diabetic neuropathy and retinopathy stratified by combined food insecurity (FI) status and Area Deprivation Index (ADI). Patients are grouped into four categories: FI with ADI ≥ 7, FI with ADI < 7, non-food-insecure (No FI) with ADI ≥ 7, and No FI with ADI < 7. ADI is categorized using state deciles (low < 7, high ≥ 7). The y-axis represents the number of patients with each complication within each subgroup. Outcomes include diabetic neuropathy and diabetic retinopathy. FI = food insecurity; ADI = Area Deprivation Index.

Additional microvascular complications of T2DM experienced by patients at the time of MACR collection within the study population, with further classification by FI and ADI groupings, are displayed in Figure 3. FI patients with an ADI ≥ 7 (*n* = 50) suffered from T2DM-related neuropathy (40.00%) and retinopathy (18.00%) (Figure 3). The prevalence of such diagnoses in FI patients with an ADI < 7 (*n* = 21) was 38.10% and 9.52% for neuropathy and retinopathy, respectively (Figure 3). The prevalence of diagnoses in food-secure patients with an ADI ≥ 7 (*n* = 57) was 36.84% and 10.53% for neuropathy and retinopathy, respectively (Figure 3). Food-secure patients with an ADI < 7 (*n* = 25) had the lowest prevalence of neuropathy (24.00%) and retinopathy (8.00%) (Figure 3). As such, FI patients with an ADI ≥ 7 showed the highest observed prevalence of neuropathy and retinopathy in this sample; however, these subgroup differences represented unadjusted associations and should be interpreted cautiously.

With further analysis, only the duration of T2DM was statistically significantly associated with neuropathy (β = 0.022, *p* = 0.040) in a linear regression after controlling for FI status, hemoglobin A1c, GFR, medication use, and ADI. Similarly, in a linear regression after controlling for the duration of T2DM, FI status, hemoglobin A1c, medication use, and ADI, only lower GFR was statistically significantly predictive of retinopathy (β = −0.006, *p* = 0.029).

**Figure 4:**
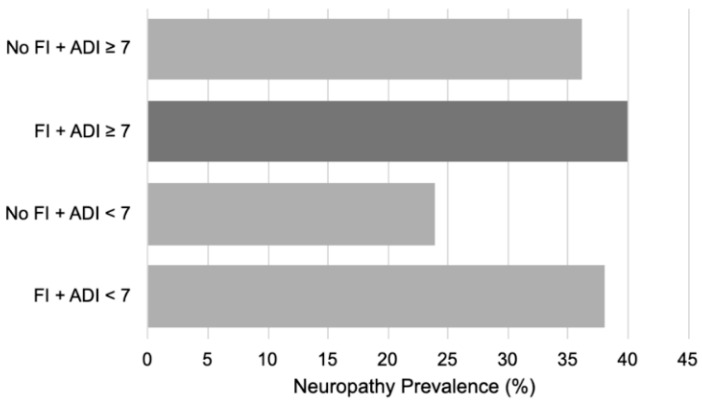
Prevalence of diabetic neuropathy (%) stratified by combined food insecurity (FI) status and Area Deprivation Index (ADI). Patients are grouped into four categories presented in the following order: non-food-insecure (No FI) with ADI ≥ 7, food-insecure (FI) with ADI ≥ 7, No FI with ADI < 7, and FI with ADI < 7. ADI is categorized using state deciles (low < 7, high ≥ 7). Neuropathy prevalence (%) is displayed for each subgroup. FI = food insecurity; ADI = Area Deprivation Index.

**Figure 5:**
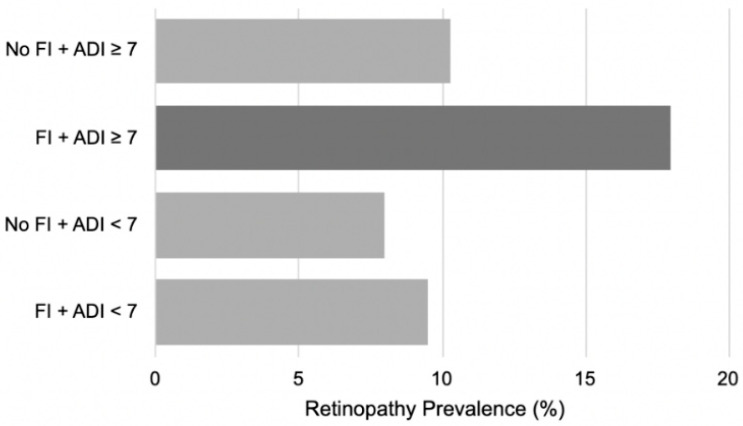
Prevalence of retinopathy (%) stratified by combined food insecurity (FI) status and Area Deprivation Index (ADI). Patients are grouped into four categories presented in the following order: non-food-insecure (No FI) with ADI ≥ 7, food-insecure (FI) with ADI ≥ 7, No FI with ADI < 7, and FI with ADI < 7. ADI is categorized using state deciles (low < 7, high ≥ 7). Retinopathy prevalence (%) is displayed for each subgroup. FI = food insecurity; ADI = Area Deprivation Index.

Interestingly, in a Chi-squared test analysis, FI participants with an ADI ≥ 7 with a T2DM duration greater than 5 years were statistically significantly more likely to have neuropathy (*X*^2^ = 5.12, *p* = 0.024, OR = 2.74, 95% CI 1.12, 6.73) and retinopathy (*X*^2^ = 10.9, *p* < 0.001). Therefore, longer T2DM duration was associated with these complications in unadjusted analyses. The prevalence of T2DM-associated neuropathy in the dataset was most significant in FI patients with an ADI ≥ 7 (40.00%), followed by patients with FI and an ADI < 7 (38.10%), food-secure patients with an ADI ≥ 7 (36.20%), and food-secure patients with an ADI < 7 (24.00%). A similar pattern was observed for patients with T2DM-associated retinopathy, with prevalence greatest in FI patients with an ADI ≥ 7 (18.00%), followed by food-secure patients with an ADI ≥ 7 (10.30%), FI with an ADI < 7 (9.50%), and food-secure patients with an ADI < 7 (8.00%). Significant results yielded in Chi-squared testing but not in linear regression suggest a difference in results between methods, which is not fully explained by the linear model for both neuropathy (Figure 4) and retinopathy (Figure 5), in which the additive effects of both FI and high ADI (≥7) yield the highest risk of microvascular complications in T2DM patients. This finding likely reflects differences between unadjusted and adjusted analyses. Accordingly, the observed subgroup differences should be interpreted cautiously, as the Chi-square findings do not account for potential confounding. The discrepancy between Chi-square and regression results reflects a difference in model structure, with Chi-square analyses representing unadjusted associations, whereas regression models adjust for confounding variables, which may attenuate observed associations here. Given the exploratory nature of these analyses and the modest sample size, these findings should be considered hypothesis-generating and require validation in larger populations.

### 3.6. Food-Secure Patients Showed Different Patterns of T2DM Pharmacotherapies

**Figure 6:**
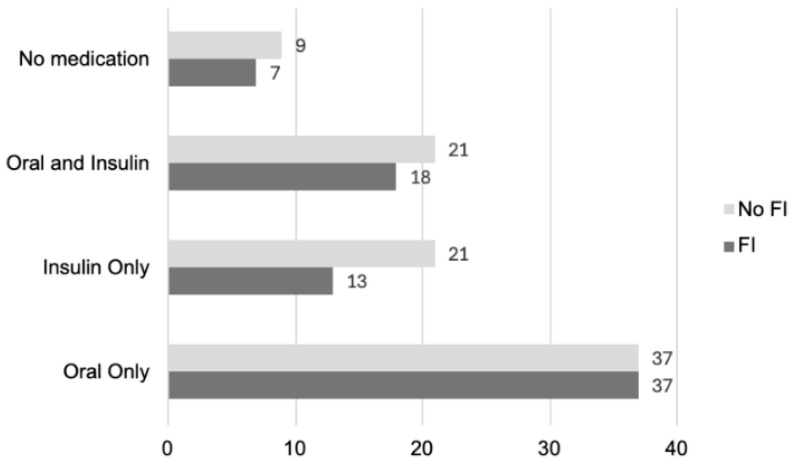
Distribution of pharmacotherapy usage (number of patients) in patients with type 2 diabetes mellitus (T2DM) stratified by food insecurity (FI) status. Medication categories are presented in the following order: no medication, oral and insulin therapy, insulin only, and oral therapy only. Patients are grouped into non-food-insecure (No FI) and food-insecure (FI) categories. Values presented are the number of patients within each FI group. Data were extracted from the electronic medical record (EMR) at the time of microalbumin-to-creatinine ratio (MACR) collection. FI = food insecurity; EMR = electronic medical record; MACR = microalbumin-to-creatinine ratio.

Another interesting aspect that relates to the progression and prevention of microvascular complications of T2DM includes pharmacotherapy utilized by patients at the time of MACR collection within the study population, which is summarized in Figure 6. The majority of both FI (37/75, 49.33%) and food-secure (37/88, 42.05%) patients used oral medication only for T2DM treatment (Figure 6). Conversely, more food-secure patients used insulin only (21/88, 23.86%) or a combination of oral and insulin therapy (21/88, 23.86%) compared with FI patients (13/75, 17.33%, and 18/75, 24.00%, respectively) (Figure 6). Classes of oral medications listed by prevalence in the dataset included: biguanides, glucagon-like peptide-1 receptor agonists, dipeptidyl peptidase-4 inhibitors, sodium-glucose-cotransporter-2 inhibitors, sulfonylureas, and thiazolidinediones. FI patients were not statistically significantly more likely to use oral medication (*X*^2^ = 1.03, *p* = 0.311, OR = 1.38, 95% CI 0.740, 2.57) or insulin alone (*X*^2^ = 0.961, *p* = 0.327, OR = 0.680, 95% CI 0.314, 1.47), or both concurrently (*X*^2^ = 0.00467, *p* = 0.946, OR = 1.03, 95% CI 0.498, 2.11) compared to food-secure patients.

## 4. Discussion

In this study, it was found that nearly half of the patients included reported experiencing FI. According to Feeding Louisiana, approximately 827,690 people, or one in six Louisianians, face the challenge of FI daily, emphasizing the importance of screening and intervention [4,5,31]. A lack of consistent access to nutritional food is especially prominent in Black, low-income communities in Louisiana [8]. This vulnerable demographic is represented by this study’s cohort, which included predominantly Black and high-ADI patients (Table 1). While this population is likely to be affected by FI, patient hesitancy in answering screening questions, misinterpretation of questions, or incomplete screening by providers may contribute to underreporting of the FI population across communities [4,31].

Potential disparities between medication access and compliance in T2DM patients experiencing FI were reflected in this dataset, as more food-secure patients managed their T2DM using insulin only or a combination of oral and insulin compared to FI patients (Figure 6). In contrast, food-secure patients with T2DM are more likely to achieve better glycemic control, suggesting better access to diabetes-friendly foods, medications, and healthcare services [32,33]. Food insecurity and neighborhood socioeconomic disadvantage may contribute to diabetic microvascular complications through different biological and behavioral pathways. Food insecurity is associated with reduced access to high-quality foods, cyclical food availability, and increased reliance on energy-dense diets. This can overall contribute to weight gain and increased chronic disease risk [34]. It has also been conceptualized as a lived stressor that can activate physiologic stress responses which promote adverse metabolic outcomes [35]. The existing literature further supports this finding as T2DM patients experiencing FI face difficulties in achieving glycemic control, leading to poor outcomes and more rapid progression of disease complications [13,32]. As glycemic control is paramount for proper T2DM management and prevention of microvascular complications, the authors expected statistically significant associations across the dataset.

In this dataset, FI alone was not a statistically significant predictor of nephropathy when comparing FI and food-secure T2DM patients ([log (MACR) t [159] = 1.14, *p* = 0.257, GFR [t [159] = −0.570, *p* = 0.570]). This aligns with the existing literature, suggesting that GFR and MACR are not acutely sensitive to FI [36,37]. However, longitudinal studies have shown that FI increases the risk of progression to ESRD over time, indicating a possible dose-dependent relationship between the time of FI exposure and the development of microvascular changes; highlighting the importance of identifying and addressing FI as part of comprehensive nephropathy management to mitigate long-term renal decline in T2DM [26]. Additionally, low diet quality in patients is associated with poor glycemic control, and this together accelerates the progression of nephropathy in T2DM patients [38,39,40,41]. Such risk is exacerbated in patients when FI coexists with high ADI and thus low socioeconomic status (SES), likely due to a lack of resources and barriers to care [32,42,43].

When adjusting for confounders, a statistically significant interaction was found between FI and ADI in predicting log (MACR) (F [1,138] = 5.07, *p* = 0.026). Patients with concomitant FI and high ADI had statistically significantly lower MACR values, indicating a lower degree of albuminuria and nephropathy compared to food-secure individuals (Figure 2), an observation that warrants further investigation, as the contrary was expected. These results may reflect FI misclassification, unmeasured social or behavioral factors (such as healthcare utilization, cultural differences, education backgrounds, and insurance status by limiting the ability for MACR screening), or factors related to healthcare access within this group. While the degree of albuminuria was less pronounced in FI patients, the interaction between FI and ADI was stronger than in food-secure patients (Figure 2). These results could suggest that ADI may exert a greater effect in FI rather than food-secure populations, demonstrating the complex interplay between FI and SES on kidney health and the multifaceted impact of adversity on diabetes management.

In the dataset, only the duration of diabetes was statistically significant in predicting neuropathy (β = 0.022, *p* = 0.040) via linear regression. However, in a Chi-squared analysis, T2DM patients were statistically more likely to develop neuropathy with an ADI ≥ 7 and a disease duration greater than 5 years (*X*^2^ = 5.12, *p* = 0.024, OR = 2.74, 95% CI 1.12, 6.73). The discrepancy in findings between the linear regression and Chi-squared analyses could be attributed to threshold effects within the dataset (Figure 4). Nonetheless, significant *X^2^* results imply that coexisting FI and high ADI (≥7) yield the highest risk of neuropathy in the study population, corroborating the combined effect of FI and SES in T2DM management. Studies regarding FI and the development of neuropathy in T2DM are limited; however, poor glycemic control and decreased intake of neuroprotective nutrients, including vitamins B6 and B12, among FI individuals is thought to increase the risk of neuropathy [44].

While one study found that MACR can predict the progression of retinopathy, a statistically significant association was not observed in this dataset, as low GFR was the only statistically significant factor [45,46,47,48]. When FI co-occurred with a high ADI, the authors observed an additive effect in the development of retinopathy—highlighting the complex interplay between SDOHs existing within the study population [49]. FI patients with high ADI were statistically more likely to develop retinopathy if they had had T2DM for 5 years or more (*X*^2^ = 10.9, *p* < 0.001). The lowest prevalence of retinopathy was seen in food-secure patients with a low ADI (Figure 5), a finding that aligns with the previous literature as these patients experience less socioeconomic disadvantage [49,50,51]. The literature reports that 24–40% of patients with poor T2DM management develop retinopathy within 5 years, with FI contributing to earlier onset and more rapid progression of retinopathy, similar to what we see in this cohort [11,52,53]. Although population-level comparison data for FI and ADI were not available, the cohort represents a health-system-based sample from a socioeconomically disadvantaged area, which may enhance relevance to a similar population while limiting broad generalizability. Additionally, some observed associations reached statistical significance with a modest effect size, which is why the interpretation of *p*-values in the context of sample size and confidence interval is important.

These findings also have an implication for healthcare policy. Integrating routine FI screenings and social determinants of health assessments could identify high-risk patients and change policy to support linkage of clinical care to nutritional services. This study focused on microvascular complications, while T2DM is associated with macrovascular complications, which could be relevant in the context of FI and socioeconomic disadvantages. Additionally, these findings may have relevance for clinical and public health practice especially when identifying high-risk patients through screening for FI and socioeconomic disadvantage. The cross-sectional design however limits the causal association.

## 5. Strengths and Limitations

This study possesses several notable strengths. Most importantly, it includes a well-characterized population with a robust representation of MACR and FI in patients with CKD and T2DM in North Louisiana. This population is underserved, with many individuals residing in areas of socioeconomic disadvantage, as indicated by ADI, and an FI rate of 16.2%, well above the national average of 12.2%, enhancing the generalizability of our findings to similarly underserved populations in the United States [54]. The observed distributions between the relationships of FI, T2DM, MACR, and complications of T2DM are consistent with the existing literature, lending external validity to these results. Furthermore, the study design enabled the examination of multiple associations across a range of clinical outcomes, providing a more nuanced understanding of the complex interplay between FI and ADI, as well as how these markers can be utilized to assess T2DM disease progression and its associated microvascular complications.

As a retrospective cohort analysis, this study is subject to inherent biases. There is a possibility of misclassification regarding the temporal relationship between diagnoses of T2DM and related microvascular complications, which may affect the interpretation of causality. The effect of FI on MACR and the development of nephropathy is not the strongest indicator of T2DM disease progression at any given point in time, although MACR is more predictive of developing kidney disease than GFR alone; however, the association with FI has not been well studied [36,37,55,56]. Additionally, despite efforts to adjust for confounders, unmeasured or residual variables may still exist, given the observational nature of the data. Relevant demographic, clinical, behavioral, healthcare-access, and social variables may also have contributed to the residual confounding in this study, as not all potentially relevant factors, such as age, sex, smoking status, glycemic control, lipid parameters, blood pressure, BMI, kidney function, diabetes duration, medication use, healthcare utilization, or other socioeconomic stress were uniformly available or incorporated into the analyses in this retrospective cohort. Missing data also posed a challenge; some patient records lacked complete clinical information, particularly among individuals who had recently relocated or whose prior medical history was inaccessible. These missing elements include, but are not limited to, dates when they were diagnosed with T2DM, when they began T2DM treatment, and often when they first developed microvascular complications. Overall, these complications led researchers to use clinical judgement to estimate when issues most likely arose in relation to MACR collection. Finally, the study sample included a limited representation of individuals of Hispanic ethnicity (Table 1), which restricts our ability to draw conclusions about the associations within this population and may limit the generalizability of our findings to more diverse demographic groups. Interestingly, the previous literature reports an extensive degree of FI and high ADI in Hispanic populations [57,58,59]. Additionally, because FI was assessed at a single time point, using the Hunger Vital Sign questionnaire, which is a validated screening tool, and not a comprehensive assessment of food insecurity, changes in status of food insecurity over time were not captured. Under-reporting related to stigma and recall bias, for example, may therefore have resulted in exposure misclassification. Additionally, given the retrospective design of this study, the findings should be interpreted as associations rather than causation. Additionally, assay-level standardization data for biochemical markers such as MACR across individual testing sites were not available in the EMR, and inter-site variation in these measurements may have influenced the assessment of microvascular outcomes.

The relatively small sample size may have also reduced the statistical power and also increased the uncertainty around the subgroup and interaction analyses. Multiple outcomes were evaluated, which may cause a type 1 error to occur. These findings should be considered exploratory and hypothesis-generating until confirmed through a larger study. Additionally, because neuropathy and retinopathy were identified from routine clinical documentation, and nephropathy was assessed using clinically available MACR values, outcome misclassification and detection bias is possible. Because of the retrospective observational nature of this design, the relationship between FI and development of microvascular complications cannot be definitively established. Because the analyses were unadjusted, potential confounding by demographic and lifestyle-related factors could not be accounted for; therefore, these findings should be interpreted cautiously. Additionally, the retrospective nature limited assessment of missing data patterns, and the evaluation of alternative ADI categorization approaches and outcomes identified through EMR documentation and ICD-10-CM codes may be influenced by differences in screening and documentation practices.

### Future Directions

Future studies should include a prospective analysis of patients to better understand the causality between observed findings. While MACR is the preferred biomarker to assess progression of kidney dysfunction, it is not acutely sensitive to FI in this dataset, despite a well-known association of worse renal outcomes with FI [26,36,37]. Future studies should utilize additional time points to assess the role of FI better in the development of these complications across a temporal span. Other measures, such as sustained elevations in HbA1c, dietary acid load, self-care behaviors, and measures of medication adherence, may also help understand the relationship between FI and microvascular complications of T2DM, specifically nephropathy, neuropathy, and retinopathy [60,61,62,63]. While there was a significant relationship between FI with concurrent high ADI and the development of retinopathy and neuropathy in this cohort, this study needs to be repeated in a larger, more diverse population to fully understand the true prevalence of this association and its broader effects on the overall health of T2DM patients. Specifically, studies aimed at understanding the driving mechanism between high ADI, FI, and worsening of microvascular complications would help guide clinical practice in managing these patients earlier in the course of their disease. Future studies may also benefit from using multilevel modeling to account for clustering of patients in neighborhoods.

## 6. Conclusions

The present investigation assessed the association between FI and ADI with development of microvascular complications in an underserved population of 163 T2DM patients in Northern Louisiana. Among study participants, 46% experienced FI, and 50.92% of patients had an ADI ≥ 7 (an average ADI of 7.71), indicating a high degree of socioeconomic disadvantage. The results suggest that FI alone was not a significant predictor of the occurrence of microvascular complications but was associated with poorer glycemic control, which may contribute to the exacerbation of these microvascular complications. When combining FI with socioeconomic disadvantage as indicated by high ADI, the odds of developing neuropathy and retinopathy were significantly higher compared to food-secure persons within the dataset. These findings should be interpreted as hypothesis-generating and indicate associations between FI, socioeconomic disadvantage, and diabetic microvascular complications, rather than causal relationships. These findings support the importance of routine food insecurity screenings. These data emphasize the importance of encouraging enhanced FI screening by healthcare providers to identify FI, ADI, and other social factors that may help inform patient-centered care strategies, especially in high-risk populations. However, additional longitudinal studies are needed to determine whether early identification or targeted interventions can reduce the burden of diabetes-related complications. Researchers encourage the replication of this study in additional populations to better understand the associations between FI and social factors in relation to T2DM management and its complications. The early identification of individuals experiencing food insecurity and socioeconomic disadvantage may help guide future intervention strategies aimed at addressing diabetes-related complications.

## Figures and Tables

**Table 1:** Demographic characteristics, smoking status, and food insecurity (FI) status of patients with type 2 diabetes mellitus (T2DM). Data were collected from the electronic medical record (EMR). “Suppressed” indicates cases with unavailable or incomplete Area Deprivation Index (ADI) data.

	FI, *n* = 75 (%)	No FI, *n* = 88 (%)	Total, *n* = 163 (%)
**Sex**			
Male	31 (41.33)	46 (52.27)	77 (47.24)
Female	44 (58.67)	42 (47.73)	86 (52.76)
**Race**			
Black or AA	41(54.67)	67 (76.14)	108 (66.26)
White	34 (45.33)	20 (22.73)	54 (33.13)
Other	0 (0.00)	1 (1.14)	1 (0.61)
**Ethnicity**			
Hispanic or Latino	0 (0.00)	1 (1.14)	1 (0.61)
Not Hispanic or Latino	75 (100.00)	87 (98.86)	162 (99.39)
**Age, Mean = 58.2 ± 10.2**			
20–30	1 (1.33)	3 (3.41)	4 (2.45)
31–40	4 (5.33)	3 (3.41)	7 (4.29)
41–50	10 (13.33)	12 (13.64)	22 (13.50)
51–60	28 (37.33)	27 (30.68)	55 (33.74)
61–70	28 (37.33)	34 (38.64)	62 (38.04)
71–75	4 (5.33)	9 (10.23)	13 (7.98)
**ADI State Decile, Mean = 7.71 ± 0.205**			
1–2	3 (4.00)	6 (6.82)	9 (5.52)
3–4	8 (10.67)	6 (6.82)	14 (8.59)
5–6	10 (13.33)	13 (14.77)	23 (14.11)
7–8	9 (12.00)	15 (17.05)	24 (14.72)
9–10	40 (53.33)	43 (48.86)	83 (50.92)
Suppressed	5 (6.67)	4 (4.55)	9 (5.52)
**Smoking Status**			
Current Smoker	15 (20.00)	12 (13.64)	27 (16.56)
Former Smoker	18 (24.00)	19 (21.59)	37 (22.70)
Never a Smoker	36 (48.00)	47 (53.41)	83 (50.92)
Unknown	1 (1.33)	7 (7.95)	8 (4.91)

**Table 2:** Descriptive mean values for microalbumin-to-creatinine ratio (MACR), hemoglobin A1c (HbA1c), estimated glomerular filtration rate (GFR), and duration of type 2 diabetes mellitus (T2DM) in patients with and without food insecurity (FI).

Metric	Mean (FI)	Mean (No FI)
MACR (mg/g)	246	225
Hemoglobin A1c (%)	8.34	8.71
GFR (mL/min/1.73 m^2^)	56	55
T2DM Duration (years)	5.05	5.37

## Data Availability

Data are available upon reasonable request from the corresponding author.

## References

[1] Gregory C.A. (2017). Food Insecurity, Chronic Disease, and Health Among Working-Age Adults.

[2] Kind A.J.H., Buckingham W.R. (2018). Making Neighborhood-Disadvantage Metrics Accessible—The Neighborhood Atlas. N. Engl. J. Med..

[3] Sharareh N., Adesoba T.P., Wallace A.S., Bybee S., Potter L.N., Seligman H., Wilson F.A. (2024). Associations between food insecurity and other social risk factors among U.S. adults. J. Gen. Intern. Med..

[4] O’Connor E.A., Webber E.M., Martin A.M., Henninger M.L., Eder M.L., Lin J.S. (2025). Preventive Services for Food Insecurity: Evidence Report and Systematic Review for the US Preventive Services Task Force. JAMA.

[5] Hunger in Louisiana. How Food Insecurity Affects Our Community. https://www.feedinglouisiana.org/hunger-in-louisiana.

[6] Hunger & Poverty in Louisiana. Map the Meal Gap. https://map.feedingamerica.org/county/2022/overall/louisiana.

[7] Hu M.D., Lawrence K.G., Bodkin M.R., Kwok R.K., Engel L.S., Sandler D.P. (2021). Neighborhood Deprivation, Obesity, and Diabetes in Residents of the US Gulf Coast. Am. J. Epidemiol..

[8] Holston D., Stroope J., Greene M., Houghtaling B. (2020). Perceptions of the Food Environment and Access among Predominantly Black Low-Income Residents of Rural Louisiana Communities. Int. J. Environ. Res. Public Health.

[9] Gold R., Kaufmann J., Gottlieb L.M., Weiner S.J., Hoopes M., Gemelas J.C., Torres C.H., Cottrell E.K., Hessler D., Marino M. (2022). Cross-Sectional Associations: Social Risks and Diabetes Care Quality, Outcomes. Am. J. Prev. Med..

[10] Gucciardi E., Vahabi M., Norris N., Del Monte J.P., Farnum C. (2014). The Intersection Between Food Insecurity and Diabetes: A Review. Curr. Nutr. Rep..

[11] ElSayed N.A., McCoy R.G., Aleppo G., Balapattabi K., Beverly E.A., Briggs Early K., Bruemmer D., Ebekozien O., Echouffo-Tcheugui J.B., American Diabetes Association Professional Practice Committee (2025). 1. Improving Care and Promoting Health in Populations: Standards of Care in Diabetes—2025. Diabetes Care.

[12] Casagrande S.S., Bullard K.M., Siegel K.R., Lawrence J.M. (2022). Food insecurity, diet quality, and suboptimal diabetes management among US adults with diabetes. BMJ Open Diabetes Res. Care.

[13] Levi R., Bleich S.N., Seligman H.K. (2023). Food Insecurity and Diabetes: Overview of Intersections and Potential Dual Solutions. Diabetes Care.

[14] Aktas G. (2025). Exploring the link: Hemogram-derived markers in type 2 diabetes mellitus and its complications. World J. Diabetes.

[15] Faselis C., Katsimardou A., Imprialos K., Deligkaris P., Kallistratos M., Dimitriadis K. (2020). Microvascular Complications of Type 2 Diabetes Mellitus. Curr. Vasc. Pharmacol..

[16] Nguyen D.V., Shaw L.C., Grant M.B. (2012). Inflammation in the pathogenesis of microvascular complications in diabetes. Front. Endocrinol..

[17] Feldman E.L., Callaghan B.C., Pop-Busui R., Zochodne D.W., Wright D.E., Bennett D.L., Bril V., Russell J.W., Viswanathan V. (2019). Diabetic neuropathy. Nat. Rev. Dis. Primers.

[18] Zhen Q., Yao N., Chen X., Zhang X., Wang Z., Ge Q. (2019). Total Body Adiposity, Triglycerides, and Leg Fat Are Independent Risk Factors for Diabetic Peripheral Neuropathy in Chinese Patients with type 2 Diabetes Mellitus. Endocr. Pract..

[19] Callaghan B.C., Xia R., Reynolds E., Banerjee M., Rothberg A.E., Burant C.F., Villegas-Umana E., Pop-Busui R., Feldman E.L. (2016). Association Between Metabolic Syndrome Components and Polyneuropathy in an Obese Population. JAMA Neurol..

[20] Solomon S.D., Chew E., Duh E.J., Sobrin L., Sun J.K., VanderBeek B.L., Wykoff C.C., Gardner T.W. (2017). Diabetic Retinopathy: A Position Statement by the American Diabetes Association. Diabetes Care.

[21] Gibson D.M. (2019). Food Insecurity, Eye Care Receipt, and Diabetic Retinopathy Among US Adults with Diabetes: Implications for Primary Care. J. Gen. Intern. Med..

[22] Ravindranath R., Bernstein I.A., Fernandez K.S., Ludwig C.A., Wang S.Y. (2023). Social Determinants of Health and Perceived Barriers to Care in Diabetic Retinopathy Screening. JAMA Ophthalmol..

[23] Feng X., Farej R., Dean B.B., Xia F., Gaiser A., Kong S.X., Elliott J., Lindemann S., Singh R. (2022). CKD Prevalence Among Patients With and Without Type 2 Diabetes: Regional Differences in the United States. Kidney Med..

[24] Rout P., Jialal I. (2025). Diabetic Nephropathy. StatPearls.

[25] Crews D.C., Kuczmarski M.F., Grubbs V., Hedgeman E., Shahinian V.B., Evans M.K., Zonderman A.B., Burrows N.R., Williams D.E., Saran R. (2014). Effect of food insecurity on chronic kidney disease in lower-income Americans. Am. J. Nephrol..

[26] Banerjee T., Crews D.C., Wesson D.E., Dharmarajan S., Saran R., Ríos Burrows N., Saydah S., Powe N.R., Powe N.R., Banerjee T. (2017). Food Insecurity, CKD, and Subsequent ESRD in US Adults. Am. J. Kidney Dis..

[27] ElSayed N.A., McCoy R.G., Aleppo G., Balapattabi K., Beverly E.A., Briggs Early K., Bruemmer D., Echouffo-Tcheugui J.B., Ekhlaspour L., American Diabetes Association Professional Practice Committee (2025). 11. Chronic Kidney Disease and Risk Management: Standards of Care in Diabetes—2025. Diabetes Care.

[28] Puchulu M.B., Garcia-Fernandez N., Landry M.J. (2023). Food Insecurity and Chronic Kidney Disease: Considerations for Practitioners. J. Ren. Nutr..

[29] Gattu R.K., Paik G., Wang Y., Ray P., Lichenstein R., Black M.M. (2019). The Hunger Vital Sign Identifies Household Food Insecurity among Children in Emergency Departments and Primary Care. Children.

[30] Rollings K.A., Noppert G.A., Griggs J.J., Melendez R.A., Clarke P.J. (2023). Comparison of two area-level socioeconomic deprivation indices: Implications for public health research, practice, and policy. PLoS ONE.

[31] Nicholson W.K., Silverstein M., Wong J.B., Chelmow D., Coker T.R., Fernandez A., Gibson E., Jaén C.R., Krousel-Wood M., US Preventive Services Task Force (2025). Screening for Food Insecurity: US Preventive Services Task Force Recommendation Statement. JAMA.

[32] Mosley-Johnson E., Walker R.J., Nagavally S., Hawks L., Bhandari S., Trasser H., Campbell J.A., Egede L.E. (2022). Relationship between food insecurity and housing instability on quality of care and quality of life in adults with diabetes. PLoS ONE.

[33] Berkowitz S.A., Meigs J.B., DeWalt D., Seligman H.K., Barnard L.S., Bright O.-J.M., Schow M., Atlas S.J., Wexler D.J. (2015). Material Need Insecurities, Control of Diabetes Mellitus, and Use of Health Care Resources: Results of the Measuring Economic Insecurity in Diabetes Study. JAMA Intern. Med..

[34] Seligman H.K., Laraia B.A., Kushel M.B. (2010). Food Insecurity Is Associated with Chronic Disease among Low-Income NHANES Participants. J. Nutr..

[35] Adler N.E., Rehkopf D.H. (2008). U.S. disparities in health: Descriptions, causes, and mechanisms. Annu. Rev. Public Health.

[36] Waikar S.S., Rebholz C.M., Zheng Z., Hurwitz S., Hsu C., Feldman H.I., Xie D., Liu K.D., Mifflin T.E., Eckfeldt J.H. (2018). Biological Variability of Estimated GFR and Albuminuria in CKD. Am. J. Kidney Dis..

[37] Viswanathan G., Sarnak M.J., Tighiouart H., Muntner P., Inker L.A. (2013). The association of chronic kidney disease complications by albuminuria and glomerular filtration rate: A cross-sectional analysis. Clin. Nephrol..

[38] Gallegos D. (2025). Effects of Food and Nutrition Insecurity on Global Health. N. Engl. J. Med..

[39] Berkowitz S.A., Karter A.J., Corbie-Smith G., Seligman H.K., Ackroyd S.A., Barnard L.S., Atlas S.J., Wexler D.J. (2018). Food Insecurity, Food “Deserts,” and Glycemic Control in Patients with Diabetes: A Longitudinal Analysis. Diabetes Care.

[40] Seligman H.K., Jacobs E.A., López A., Tschann J., Fernandez A. (2012). Food Insecurity and Glycemic Control Among Low-Income Patients with Type 2 Diabetes. Diabetes Care.

[41] Berkowitz S.A., Baggett T.P., Wexler D.J., Huskey K.W., Wee C.C. (2013). Food Insecurity and Metabolic Control Among U.S. Adults with Diabetes. Diabetes Care.

[42] Kurani S.S., Lampman M.A., Funni S.A., Giblon R.E., Inselman J.W., Shah N.D., Allen S., Rushlow D., McCoy R.G. (2021). Association Between Area-Level Socioeconomic Deprivation and Diabetes Care Quality in US Primary Care Practices. JAMA Netw. Open.

[43] Kurani S.S., Heien H.C., Sangaralingham L.R., Inselman J.W., Shah N.D., Golden S.H., McCoy R.G. (2022). Association of Area-Level Socioeconomic Deprivation with Hypoglycemic and Hyperglycemic Crises in US Adults with Diabetes. JAMA Netw. Open.

[44] Lee J.S., Frongillo E.A. (2001). Nutritional and Health Consequences Are Associated with Food Insecurity Among U.S. Elderly Persons. J. Nutr..

[45] Chen Y.-H., Chen H.-S., Tarng D.-C. (2012). More Impact of Microalbuminuria on Retinopathy Than Moderately Reduced GFR Among Type 2 Diabetic Patients. Diabetes Care.

[46] Man R.E.K., Sasongko M.B., Wang J.J., MacIsaac R., Wong T.Y., Sabanayagam C., Lamoureux E.L. (2015). The Association of Estimated Glomerular Filtration Rate with Diabetic Retinopathy and Macular Edema. Investig. Ophthalmol. Vis. Sci..

[47] Kong A.P.S., So W.Y., Szeto C.C., Chan N.N., Luk A., Ma R.C.W., Ozaki R., Ng V.W.S., Ho C.S., Lam C.W.K. (2006). Assessment of glomerular filtration rate in addition to albuminuria is important in managing type II diabetes. Kidney Int..

[48] Mottl A.K., Kwon K.S., Garg S., Mayer-Davis E.J., Klein R., Kshirsagar A.V. (2012). The association of retinopathy and low GFR in type 2 diabetes. Diabetes Res. Clin. Pract..

[49] Tan L., Wang J., Han J., Sainsbury C., Denniston A.K., Crowe F.L., Toulis K.A., Karamat M.A., Yao M., Nirantharakumar K. (2024). Socioeconomic Deprivation and the Risk of Sight-Threatening Diabetic Retinopathy: A Population-Based Cohort Study in the U.K. Diabetes Care.

[50] Low L., Law J.P., Hodson J., McAlpine R., O’Colmain U., MacEwen C. (2015). Impact of socioeconomic deprivation on the development of diabetic retinopathy: A population-based, cross-sectional and longitudinal study over 12 years. BMJ Open.

[51] Giannakis P., Nderitu P., Nunez Do Rio J.M., Webster L., Mann S., Hopkins D., Cardoso M.J., Modat M., Bergeles C., Jackson T.L. (2024). Effect of socioeconomic deprivation as determined by the English deprivation deciles on the progression of diabetic retinopathy and maculopathy: A multivariate case–control analysis of 88 910 patients attending a South-East London diabetic eye screening service. Br. J. Ophthalmol..

[52] Flaxel C.J., Adelman R.A., Bailey S.T., Fawzi A., Lim J.I., Vemulakonda G.A., Ying G. (2020). Diabetic Retinopathy Preferred Practice Pattern^®^. Ophthalmology.

[53] ElSayed N.A., McCoy R.G., Aleppo G., Balapattabi K., Beverly E.A., Briggs Early K., Bruemmer D., Callaghan B.C., Echouffo-Tcheugui J.B., American Diabetes Association Professional Practice Committee (2025). 12. Retinopathy, Neuropathy, and Foot Care: Standards of Care in Diabetes—2025. Diabetes Care.

[54] Rabbitt M.P., Reed-Jones M., Hales L.J., Burke M.P., United States Department of Agriculture Economic Research Service (2024). Household Food Security in the United States in 2023.

[55] Gansevoort R.T., Nauta F.L., Bakker S.J. (2010). Albuminuria: All you need to predict outcomes in chronic kidney disease?. Curr. Opin. Nephrol. Hypertens..

[56] Levey A.S., Becker C., Inker L.A. (2015). Glomerular Filtration Rate and Albuminuria for Detection and Staging of Acute and Chronic Kidney Disease in Adults: A Systematic Review. JAMA.

[57] López M.Á., Fuster M., Fleckman J., George A., Chaparro M.P. (2024). Time-Trends in Food Insecurity Among US-Born and Foreign-Born Hispanic Adults by Language Use: An Analysis of National Health and Nutrition Examination Survey Data, 1999–2018. J. Acad. Nutr. Diet..

[58] Varela E.G., McVay M.A., Shelnutt K.P., Mobley A.R. (2023). The Determinants of Food Insecurity Among Hispanic/Latinx Households with Young Children: A Narrative Review. Adv. Nutr..

[59] Driver N., Tebbe M., Burke M., Amin N.S. (2023). Factors associated with food insecurity among Latinx/Hispanics in the U.S.: Evidence from the Fragile Families & Childhood Wellbeing Study. Ethn. Health.

[60] Heerman W.J., Wallston K.A., Osborn C.Y., Bian A., Schlundt D.G., Barto S.D., Rothman R.L. (2016). Food insecurity is associated with diabetes self-care behaviours and glycaemic control. Diabet. Med..

[61] Schroeder E.B., Zeng C., Sterrett A.T., Kimpo T.K., Paolino A.R., Steiner J.F. (2019). The longitudinal relationship between food insecurity in older adults with diabetes and emergency department visits, hospitalizations, hemoglobin A1c, and medication adherence. J. Diabetes Complicat..

[62] DeJesus R.S., Grimm J.A., Fan C., Sauver J.S. (2024). Exploring the association of social connections and food security among adults with uncontrolled type 2 diabetes: A population-based study. J. Health Popul. Nutr..

[63] Della Guardia L., Thomas M., Cena H. (2018). Insulin Sensitivity and Glucose Homeostasis Can Be Influenced by Metabolic Acid Load. Nutrients.

